# Centronuclear Myopathy in Labrador Retrievers: A Recent Founder Mutation in the *PTPLA* Gene Has Rapidly Disseminated Worldwide

**DOI:** 10.1371/journal.pone.0046408

**Published:** 2012-10-05

**Authors:** Marie Maurer, Jérôme Mary, Laurent Guillaud, Marilyn Fender, Manuel Pelé, Thomas Bilzer, Natasha Olby, Jacques Penderis, G. Diane Shelton, Jean-Jacques Panthier, Jean-Laurent Thibaud, Inès Barthélémy, Geneviève Aubin-Houzelstein, Stéphane Blot, Christophe Hitte, Laurent Tiret

**Affiliations:** 1 CNM Project, Université Paris-Est Créteil, Ecole Nationale Vétérinaire d'Alfort, Maisons-Alfort, France; 2 UMR955 de Génétique Fonctionnelle et Médicale, Institut National de la Recherche Agronomique, Maisons-Alfort, France; 3 Antagene, La Tour de Salvagny, France; 4 CNM Project, Pickett, Wisconsin, United States of America; 5 Institut für Neuropathologie, Heinrich-Heine-Universität, Düsseldorf, Germany; 6 College of Veterinary Medicine, Neurology Faculty, North Carolina State University, Raleigh, North Carolina, United States of America; 7 College of Medical, Veterinary and Life Science, School of Veterinary Medicine, University of Glasgow, Glasgow, United Kingdom; 8 Department of Pathology, University of California San Diego, La Jolla, California, United States of America; 9 Mouse Functional Genetics URA2578, Centre National de la Recherche Scientifique, Institut Pasteur, Paris, France; 10 Unité Propre de Recherche de Neurobiologie, Université Paris-Est Créteil, Ecole Nationale Vétérinaire d'Alfort, Maisons-Alfort, France; 11 UMR6290, Centre National de la Recherche Scientifique, Institut de Génétique et Développement de Rennes, Université de Rennes1, Rennes, France; Institut Jacques Monod, France

## Abstract

Centronuclear myopathies (CNM) are inherited congenital disorders characterized by an excessive number of internalized nuclei. In humans, CNM results from ∼70 mutations in three major genes from the myotubularin, dynamin and amphiphysin families. Analysis of animal models with altered expression of these genes revealed common defects in all forms of CNM, paving the way for unified pathogenic and therapeutic mechanisms. Despite these efforts, some CNM cases remain genetically unresolved. We previously identified an autosomal recessive form of CNM in French Labrador retrievers from an experimental pedigree, and showed that a loss-of-function mutation in the protein tyrosine phosphatase-like A (*PTPLA*) gene segregated with CNM. Around the world, client-owned Labrador retrievers with a similar clinical presentation and histopathological changes in muscle biopsies have been described. We hypothesized that these Labradors share the same *PTPLA^cnm^* mutation. Genotyping of an international panel of 7,426 Labradors led to the identification of *PTPLA^cnm^* carriers in 13 countries. Haplotype analysis demonstrated that the *PTPLA^cnm^* allele resulted from a single and recent mutational event that may have rapidly disseminated through the extensive use of popular sires. *PTPLA*-deficient Labradors will help define the integrated role of *PTPLA* in the existing CNM gene network. They will be valuable complementary large animal models to test innovative therapies in CNM.

## Introduction

In humans, myotubular/centronuclear myopathies, often referred to as CNM, are congenital inherited myopathies characterized by generalized muscle weakness associated with respiratory insufficiency, external ophthalmoplegia and normal function of the central and peripheral nervous system. Muscle biopsies show a type 1 fiber predominance and excessive numbers of fibers with internalized or centralized nuclei [1], [2]. Clinical presentations in patients are very heterogeneous and in most instances, correlate with mutations in distinct genes. The very severe X-linked form (XLMTM, OMIM 310400) affects neonates and carries a poor prognosis. This form is due to mutations in the myotubularin gene (*MTM1*; www.hgmd.cf.ac.uk and [3]). Milder late-onset childhood or adult-onset autosomal dominant forms (ADCNM, OMIM 160150) are mainly due to mutations in the dynamin 2 gene (*DNM2*) or, in one reported case, in the ryanodine receptor gene (*RYR1*) [4], [5]. Intermediate autosomal recessive forms (ARCNM, OMIM 255200) are due to mutations in the BIN1/amphiphysin 2 (*BIN1*), myotubularin-related 14 (*MTMR14*) [6], [7] or *RYR1* genes [8], [9]. Despite these major advances in the identification of CNM-causing genes in humans, 30% of sporadic or familial cases remain genetically unresolved, underlying the existence of additional causative genes in the CNM functional network.

Years ago, an autosomal recessive congenital canine CNM was described in Labradors from an experimental pedigree developed in France from two probands [10], [11]. By linkage analysis, the locus was mapped to canine chromosome 2, and an associated mutation was identified in a gene annotated as the protein tyrosine phosphatase-like A (*PTPLA*) gene. In affected dogs, the homozygous genotype resulting from the insertion of a SINE within exon 2 of *PTPLA* correlated with a complex panel of splicing defects in skeletal muscles, eventually leading to a 99% decrease in the amount of wild-type *PTPLA* transcripts [12], compatible with a loss-of-function mutation. For decades, phenotypically similar myopathies have been reported in client-owned Labradors living in the USA, the United Kingdom, Australia, Canada and Europe [13], [14], [15], [16], [17], and have been named type II fiber deficiency [13], autosomal recessive muscular dystrophy [18] or hereditary myopathy of Labrador retrievers (HMLR) [19], [20]. Here we demonstrate that regardless of the country of origin, every client-owned Labrador retriever diagnosed with any of these phenotypically similar myopathies carried the same *PTPLA* loss-of-function allele first identified in our experimental pedigree. Further, our findings provide evidence that this allele originated from a leading founder that sustained rapid dissemination worldwide. Finally, we show that the variable expression of disease severity in affected dogs does not rely on genetic polymorphisms within the inserted SINE sequence.

## Results

### Selection of an international panel of CNM/Phenotypically similar Labrador retrievers

**Figure 1:**
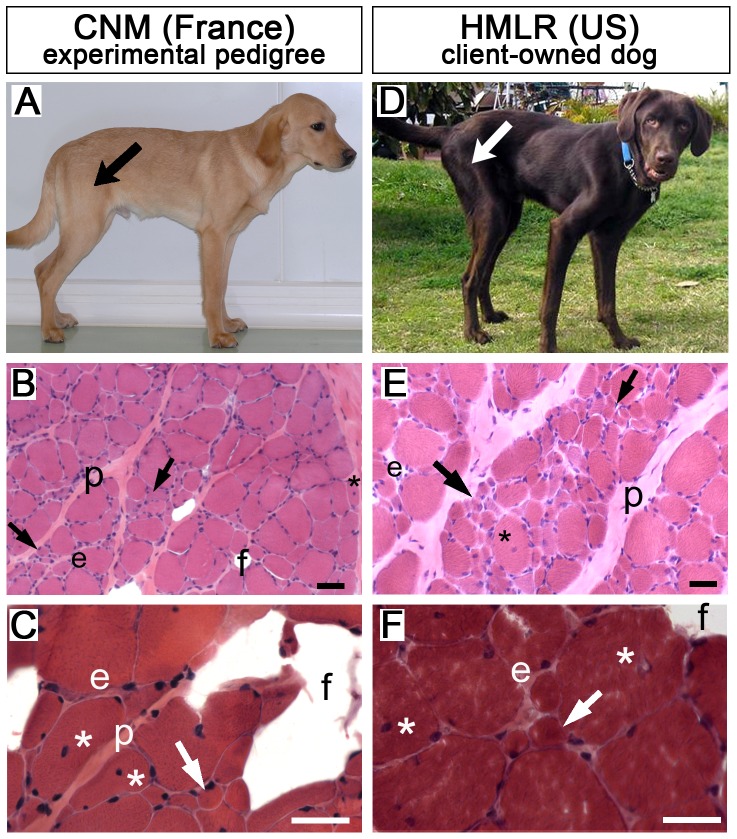
Client-owned US Labradors share similar morphological and histopathological features with French CNM dogs from the experimental pedigree. French CNM (A–C) and US HMLR (D–F) affected dogs have atrophic skeletal muscles, the most affected being those of pelvic limbs (e.g. *biceps femoris* muscle, arrows in A,D). B,C,E,F are Hematoxylin-Eosin-stained transverse sections of the *biceps femoris* muscle from 6-month-old (B, FR-4; E, US-18) or 10-year-old (C,F) affected Labradors. Early signs include groups of atrophic fibers, surrounded by endomysial (e) and perimysial (p) fibrosis. In older dogs, increased internalized or centralized nuclei (asterisks) and fatty infiltration (f) are observed. Scale bar = 50 µm.

To perform a global genetic analysis on CNM/Phenotypically similar dogs, further referred to as CNM Labradors, we set up an initial confirmation panel of DNA from 32 client-owned Labradors living in the USA, Germany, the UK, France and Denmark, which had been initially diagnosed with type 2 fiber deficiency, autosomal recessive muscular dystrophy, HMLR or CNM (Table 1, Table 2 and Table S1). Two Labradors with a diagnosis of myasthenia gravis or primary neuropathy were included as controls. Although records were incomplete in some cases, clinical signs in affected dogs included gait abnormalities, generalized weakness, fatigability, absence of patellar reflexes, and generalized muscle atrophy prominently affecting limb, cervical and temporal muscles. Structural remodeling of skeletal muscles included atrophic (≤25 µm of diameter) and anguloid-round fibers, fiber size variation, endomysial and perimysial fibrosis, predominance of type I fibers and internalization or centralization of nuclei in some fibers (Figure 1 and Table S1).

**Table 1:** Number of genotyped dogs used in this study.

	+/+	+/*cnm*	*cnm*/*cnm*	Total
Dogs tested for the mutation *(whole panel)*	6 173	1 173	80	7 426
Dogs with clinical or histopathological reports *(initial confirmation panel)*	5	1	26	32
Dogs included in the haplotype analysis	39	-	32	71
Dogs sequenced (SINE insertion)	-	-	12	12

The whole panel includes all dogs for which samples were received for testing purposes. The initial confirmation panel includes dogs with an early diagnosis of HLMR or phenotypically similar myopathies (Table S1).

**Table 2:** Numbers by genotype of Labradors diagnosed with HMLR or phenotypically similar myopathy.

	+/+	+/*cnm*	*cnm*/*cnm*	Total
US	0	1	14	15
Germany	4	0	7	11
UK	1	0	2	3
France	0	0	2	2
Denmark	0	0	1	1
**Total**	**5**	**1**	**26**	**32**

Countries of origin of dogs are listed. With the exception of two German Labradors used as controls, all Labradors were genotyped because they had initially been diagnosed with HMLR or phenotypically similar myopathies (Table S1).

### A unique mutation in CNM Labradors

**Figure 2:**
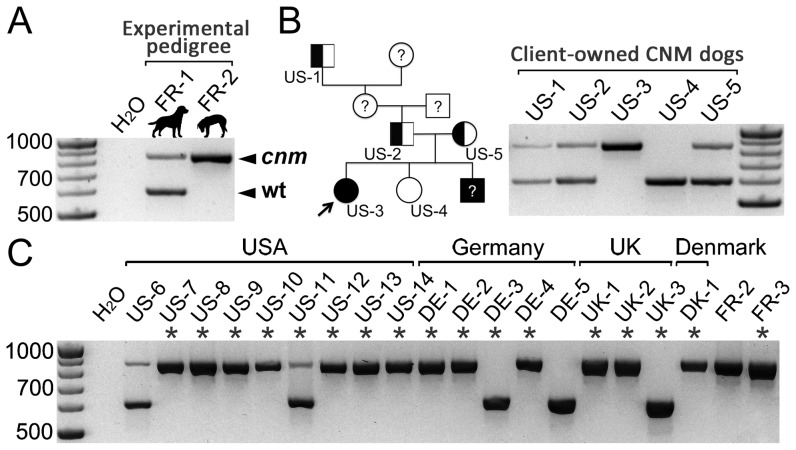
*PTPLA* mutation in the initial confirmation panel of CNM dogs. (A) Wild-type (wt = 610 bp) and *PTPLA^cnm^* (*cnm* = wt+238 bp) alleles in a healthy carrier (FR-1) and an affected (FR-2) Labrador from the French experimental pedigree. (B) Segregation of the *PTPLA^cnm^* allele in a four-generation pedigree of a client-owned US proband female (arrow). (C) Genotypes of client-owned Labradors from several countries, diagnosed with CNM-related myopathies (asterisks; Table S1). US-6 is a champion known to have produced CNM pups; DE-5 is a control affected by a neuropathy and FR-2 was reloaded for size comparison.

It was previously shown that Labradors from a French experimental pedigree segregating CNM carry two copies of the *PTPLA* g.9459-9460ins238 mutation (Figure 2A; [12]). First, to determine whether client-owned CNM Labradors from the USA carry the same recessive disease-causing mutation, one affected female proband from the initial confirmation panel (US-3), an unaffected sister, parents, and a great-grandfather were genotyped by PCR, as described [12]. DNA from the healthy sister yielded a unique product of 610 bp, which is the size of the wild-type *PTPLA* allele. In contrast, DNA from the female proband yielded a unique product of ∼850 bp, a size corresponding to the CNM-causing allele. The two healthy parents and great-grandfather were heterozygotes (Figure 2B).

Second, genotypes of the 32 dogs from the initial confirmation panel were analyzed (Figure 2C and full list of results in Table S1). We confirmed that the two control dogs were homozygous for the wild-type allele (DE-3 and DE-5), and identified that 77% of dogs (23/30) were homozygous for the *PTPLA* mutation. One was heterozygous and six were homozygous for the wild-type allele. The heterozygous USA dog (US-11) expressed none of the early histopathological signs of CNM, but a type 1 predominance that has never been observed in heterozygous dogs from the experimental pedigree. An idiopathic etiology was favored for this dog. Wild-type dogs were from the UK and Germany and corresponded to orphan cases for which precise clinical or histopathological records were missing.

**Figure 3:**
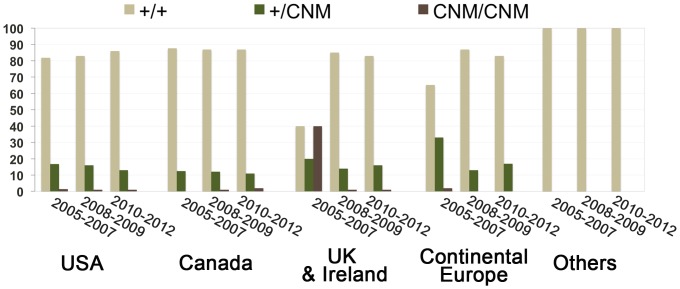
Percentage of wild-type homozygous (+/+), healthy carriers (+/*cnm*) and CNM affected (*cnm*/*cnm*) Labradors tested for medical or breeding purposes. The total number of dogs for each period is indicated above histograms. Additional dog samples from Australia (n = 15), New-Zealand (n = 2), Puerto Rico (n = 1) and Argentina (n = 1) were tested; they were all homozygous for the wild-type allele (+/+).

A genetic test was thus proposed to owners for diagnostic or breeding purposes (www.labradorcnm.com). In the last 7 years, we received and genotyped samples from 7,426 Labradors living in 18 countries (Figure 3 and Table 3). In this unique comprehensive panel of client-owned dogs, we identified 80 dogs from six countries that were homozygous for the mutated *PTPLA* allele. Sixty-eight were young dogs that had already displayed clinical signs of CNM and twelve were asymptomatic one-month-old pups at the time of testing; a few weeks after testing, all pups displayed clinical signs consistent with CNM. On the contrary, none of the 1,172 heterozygous dogs living in 13 different countries displayed clinical signs of CNM, confirming the strict autosomal recessive mode of inheritance of CNM in Labradors. Affected and healthy carriers included both males and females with the three recognized yellow, black and chocolate coat colors (Table S2 and Table S3).

**Table 3:** Numbers by genotype of Labradors tested for medical or breeding purposes.

	2005–2007			2008–2009			2010–2012			
	+/+	+/*cnm*	*cnm*/*cnm*	+/+	+/*cnm*	*cnm*/*cnm*	+/+	+/*cnm*	*cnm*/*cnm*	Total
US	1 954	400	32	1 575	302	19	1 195	186	9	5 672
*– carriers*		*16,8%*			*15,9%*			*13,4%*		
Canada	155	22	0	103	14	1	104	13	2	414
*– carriers*		*12,4%*			*11,9%*			*10,9%*		
UK & Ireland	10	5	10	253	41	2	141	28	1	491
*– carriers*		*20%*			*13,9%*			*16,5%*		
Continental Europe	140	71	4	324	50	0	201	40	0	830
*– carriers*		*33%*			*13,4%*			*16,6%*		
Others	5	0	0	7	0	0	7	0	0	19
Total	2 264	498	46	2 262	407	22	1 648	267	12	7 426

Dogs are grouped by geographical origin and period of testing. The percentage of healthy carriers (+/*cnm*), suggestive of the *PTPLA^cnm^* allele segregation in Labrador lines, is provided.

Reports of clinical signs in the 80 CNM genotyped dogs or of histopathological features in affected dogs from the initial confirmation panel suggested a spectrum in severity of the disease (Table S1). In the *SILV/PMEL* gene, the length of the oligo(dA)-rich tail of an inserted SINE was shown to influence the merle phenotype penetrance in dogs [21]. Thus, we visually checked the size of the *PTPLA^cnm^* allele amplified from DNAs of the 1,172 healthy carriers from the whole panel and the 80 affected dogs (representative panel in Figure S1). No fragment length polymorphisms were observed. The *PTPLA^cnm^* allele was further sequenced in 12 CNM dogs from the initial confirmation panel and no base pair polymorphisms were identified within SINE sequences (Figure S2).

In light of the finding that all affected Labradors share a unique well-conserved mutation causing muscle defects analogous to those seen in human forms of CNM, we propose that the mutated allele, initially named *PTPLA^alf^* [12], becomes *PTPLA^cnm^*.

### Founder effect for the *PTPLA^cnm^* allele

A unique origin for the *PTPLA^cnm^* allele was suggested by pedigree analyses showing that some affected Labradors from Germany and France had UK champions in their background (Table S1), where the prevalence of CNM is one of the highest (Table 3). To confirm that all occurrences of the disease were due to a single ancestral mutation, we constructed a series of haplotypes with SNPs from a 9-Mb region surrounding the *PTPLA* locus (Table S4 and Table S5) for each of 39 homozygous *PTPLA* ^+/+^ healthy Labradors and 32 *PTPLA^cnm^* ^/*cnm*^ affected Labradors.

**Figure 4:**
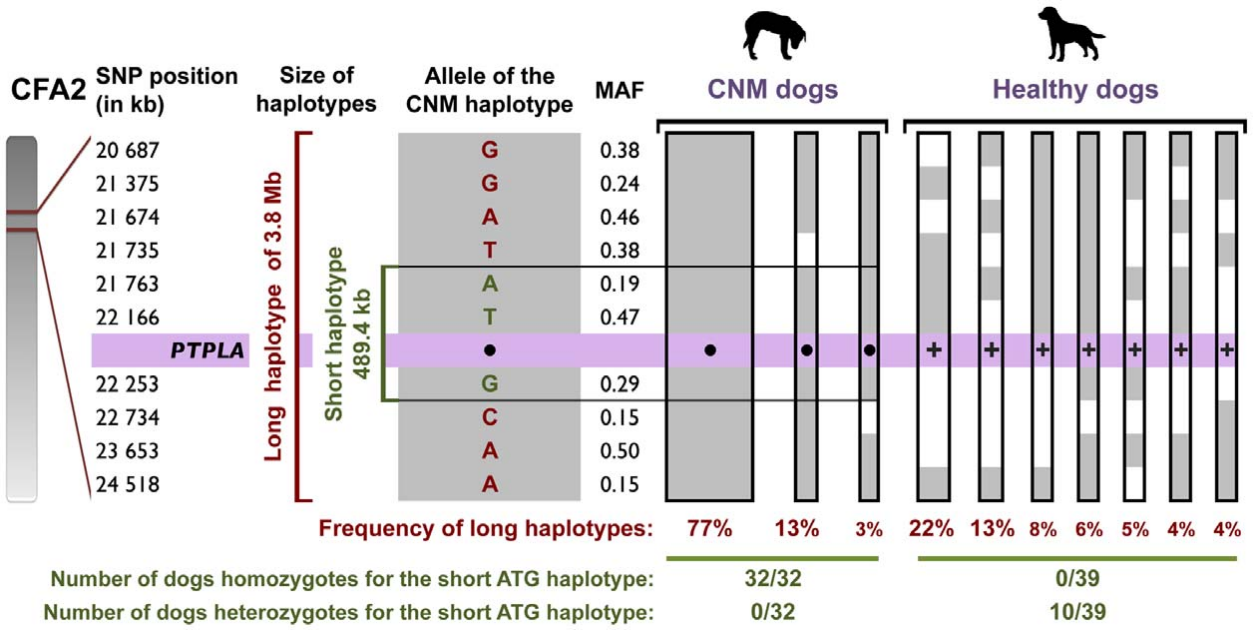
A 3.8-Mb haplotype is highly associated with CNM. The acrocentric region of the *PTPLA* locus within canine chromosome 2 (CFA2) is depicted. Positions of genotyped SNPs are indicated. The short and long haplotypes associated with CNM are shown in green and red, respectively. For each SNP, the allele detected in the CNM associated haplotype is indicated and represented as a grey box. The alternative allele is represented as a white box. For each SNP, the minor allele frequency (MAF) in the healthy population of Labradors is given. The *PTPLA^cnm^* allele is represented by a black dot (•) and the wild-type *PTPLA^+^* allele by a “+”. Frequencies of long 3.8-Mb haplotypes in each population of CNM or healthy dogs are given below each haplotype. For haplotypes with frequencies >10%, width of haplotypes is proportional to its frequency. Haplotypes with frequencies below 3% have been omitted and are detailed in Figure S3.

A thorough analysis of genotyping data revealed that 100% of affected Labradors (n = 32) shared two copies of a common short haplotype of 489.4 kb, extending from SNP 21763 to SNP 22253 (Figure 4, Figure S3). The A-T-G short haplotype is highly predictive of the disease condition in the *PTPLA^cnm^* ^/*cnm*^ affected dogs (*P* = 3.87×10^−21^), and was identified at a carrier frequency of 12.8% (10/78 haplotypes) in healthy control dogs. Further analyses indicated that a second long haplotype of 3.8 Mb and covering 10 SNPs from SNP 20687 to SNP 24518 (G-G-A-T-A-T-G-C-A-A), remained highly associated with the disease (*P* = 2.59×10^−19^). The frequency of this longer haplotype segment in affected dogs was 77,4% (48/62 haplotypes) and was not found in healthy dogs (0/78 haplotypes).

**Figure 5:**
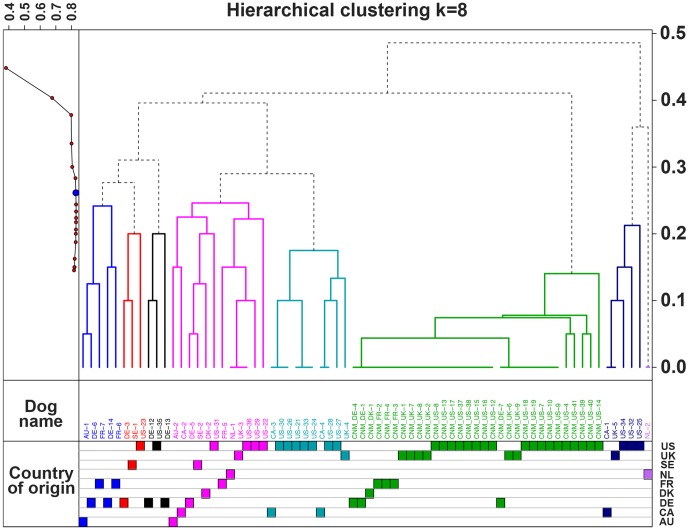
Hierarchical clustering from the 81 dogs at k = 8. The analysis was based on genotypes obtained for ten loci (SNP 20687 to SNP 24518). Hubert Gamma values are indicated for k≥2 on the top left panel. The scale on the right axis represents the genetics distances calculated by PLINK software. In the dendogram, each vertical line represents a dog and colors reflect the eight clusters obtained by the analysis. Grey dash lines indicate common ancestors inferred from the analysis. Below the dendogram, dogs are named by their unique identifier. The “CNM_” prefix was added to the name of affected Labradors.

A hierarchical clustering analysis using genotypes obtained for these 10 SNPs confirmed that at k = 8, all affected dogs segregated in a single highly predictive haplogroup, regardless of their geographic origin (Figure 5). Closer examination of this long haplotype segment revealed that CNM dogs finely stratified into distinct sub-groups, each diverging from the CNM haplotype by loss of homozygosity at one or two SNPs (Figure S3). Using the mutation-rate of 3.0×10^−8^ mutations/nucleotide/generation reported in humans [22], we calculated that 17.5 generations would separate today's Labradors from the original *PTPLA^cnm^* founder. Assuming a generation time of 2.5–3 years in dogs, we estimate that the *PTPLA^cnm^* mutation arose ∼50 years ago. Healthy Labradors were later subdivided into seven haplogroups, reflecting the genetic heterogeneity of the Labrador breed, the most popular in the world.

## Discussion

### *PTPLA^cnm^*, a prevalent and fully penetrant mutation with variable expressivity

This report shows that the *PTPLA^cnm^* allele, initially identified in a French experimental pedigree, is the worldwide fully penetrant allele causing CNM in client-owned Labradors. On the basis of the results obtained in the past 24 months, we estimate that one dog in seven is a CNM carrier (245/1,757 = 13,9%), with the highest percentages found in the UK (19%), the USA (13%) and Canada (11,5%). A high percentage of carriers in the UK (22%) has also been independently reported [23]. To date, the autosomal recessive CNM is the most prevalent hereditary myopathy segregating in Labradors.

Variable phenotypic expression of *PTPLA^cnm^* was revealed by differences in pups' gain of weight, age of first clinical signs or extent of muscle atrophy and remodeling. A first hypothesis relied on a variable *PTPLA* function resulting from a SINE-dependent modulation of the splicing machinery. For example, Merle (*SILV^M^*) is a coat color mutation that is inherited in an autosomal, incompletely dominant fashion, with rare *SILV^M/+^* dogs not exhibiting the merle phenotype. These phenotypic reversions are due to a ∼30 bp shortening of the oligo(dA)-rich tail of the SINE responsible for the *SILV^M^* mutation [21]. In CNM dogs, this mechanism can be excluded because no polymorphisms were detected in the inserted SINE. The variable phenotypic expression may thus depend upon undetected functional polymorphisms yet to be identified in *PTPLA*, its functionally redundant paralogs, or additional genetic modifiers.

### Identifying the origin of the *PTPLA^cnm^* allele

The Labrador breed is characterized by a 785-kb linkage disequilibrium, the shortest in dogs [24]. The simplest explanation for the presence of a long 3.8 Mb haplotype segment shared by CNM affected dogs, is that the ancestral *PTPLA^cnm^* allele arose recently, about 50 years ago, but sufficiently long ago for the accumulation of *de novo* mutations. The large size of the CNM haplotype and the high percentage of carriers favor a rapid expansion of the haplotype among Labradors, suggesting that the mutation appeared within the pedigree of a very famous stud. Accordingly, several National Stake winners of the 1950s and the 1970s are dominant in pedigrees of contemporaneous champion dogs [25], and the first clinical description has been reported in 1976 [13]. Absence of the associated haplotype in the tested healthy population highly suggests that this champion emerged from a marginal line of Labradors, or resulted from introduction of genetic diversity from other breeds.

### CNM Labradors, a model in comparative pathophysiology and therapeutics

A global therapeutic strategy for this heterogeneous group of CNM may be economically relevant and to reach this goal, it is essential to understand how the different CNM-causing genes, including *PTPLA*, interact to build a functional muscle and to maintain its homeostasis. A collection of animal models with loss- or gain-of-function mutations in CNM-causing genes has been developed. They have been instrumental in identifying common defects in membrane organization, trafficking or remodeling (reviewed in [26], [27]), mimicking structural aberrations observed in muscle biopsies from *BIN1*, *MTM1*, and *DNM2*-CNM patients [28], [29]. In humans and CNM animal models, altered triad junctional complexes have been observed, suggesting deficient intracellular Ca^2+^ homeostasis and impaired excitation-contraction coupling [28], [30], [31], [32], [33]. A plausible mechanism is that DNM2 at the Z-disk would play a role in the transverse orientation of T-tubules through its interaction with BIN1, localized at the T-tubule [34]; the concomitant recruitment of DNM2 and BIN1, two phosphoinositide-binding proteins, would be tightly regulated by the phospahtidylinositol (PtdIns) 3-phosphatase activity of MTM1 and MTMR14 [7], [35]. Once established, excitation-contraction complexes would be maintained and functionally regulated by CNM genes. Indeed deregulation of the Ca^2+^ handling in adult CNM muscles have been attributed to increased PtdIns(3,5)*P*2 levels on the activity of the RYR1 Ca^2+^ sarcoplasmic channel [32], or to a PtdIns-independent consequence of MTM1 deficiency on mitochondrial positioning and homeostasis [29]. Abnormal membrane traffic at the neuromuscular junction has also been shown in MTM patients [36] and mice models [37]. Finally, it has been shown that deficiency of MTMR14 alone, or in combination with MTM1, promotes autophagy initiation through increased levels of PtdIns3*P*, thereby suggesting that the CNM pathomechanism is complex and may combine regulation of intracellular Ca^2+^ homeostasis, neuromuscular junction efficiency and autophagy.

PTPLA is a 3-hydroxyacyl-CoA dehydratase (HACD), which is an endoplasmic reticulum resident enzyme that catalyzes the third reaction of elongation of very long chain fatty acids (VLCFA) [38], [39]. Saturated and monounsaturated VLCFA are components of sphingolipids, a large family of lipids that are enriched in lipid rafts and display crucial structural and signaling roles [40]. Directly or following their inclusion into sphingolipids and phosphoinositides, VLCFA may participate in muscle homeostasis by targeting phosphoinositides to specific cellular compartments or by regulating their levels. Indeed deficiency in the yeast *PTPLA* ortholog (*Phs1*) decreases the amount of VLCFA and corresponding sphingolipids and indirectly reduces the level of some phosphoinositides [38], [41]. A complementary role of VLCFA may be to promote the clustering of neuromuscular junction components and signaling complexes in lipid rafts. In-depth analysis of PTPLA-deficient animals will help precisely understand the role of VLCFA in the functional network of other CNM genes. Ultimately, CNM Labradors will be a relevant large animal model for inclusion in pre-clinical trials of innovative drugs and gene or cell therapies.

## Materials and Methods

### Dogs Included in the Study

A total of 7,426 Labrador retrievers from 19 countries were included in the study (whole panel, Table 1). A subgroup of the whole panel was named the “international confirmation panel”. It was composed of 32 dogs from the US, Germany, UK, Denmark and France for which clinical, histopathological or genetic reports were provided (Table 2, Table S1). Haplotype analyses were conducted on a representative group of 71 healthy (wild-type, +/+) or affected (CNM, *cnm*/*cnm*) Labradors from 9 countries. Each dog was assigned a unique identifier made of a 2-letter code for its country of origin, followed by an incremental number (e.g. UK-3).

### Ethics statement

All but one dog, FR-2, were examined with the consent of their owners. Blood and biopsies were obtained as part of routine clinical procedures for diagnostic purposes. Cheek cells were collected by owners or veterinarians using non-invasive swabs. As the data were from client-owned dogs undergoing normal veterinary exams, there was no “animal experiment” according to the legal definitions in France, Germany, Denmark, the US and the UK. All local regulations related to clinical procedures were observed.

FR-2 was a founder dog of our experimental pedigree, and materials from FR-2 used in this study were frozen samples obtained in the 1990s by one of the co-authors (SB). At the time FR-2 was sampled, there was no animal welfare committee at the Ecole nationale vétérinaire d'Alfort; however, SB was accredited by the Veterinary Division of the French Ministry of Agriculture to perform research on animals.

### DNA Extraction and SINE Sequence Analysis

Genomic DNA was extracted either from blood using a proteinase K digestion followed by a classical phenol/chloroform purification or from cheek swabs using the ChargeSwitch© gDNA buccal cell kit following manufacturer's instructions (Invitrogen).

SINE amplification by PCR was carried out as previously described [12], using the following primers: 5′-CCTCGAAGAAGGGTCAGTGTAA-3′ and 5′-CCAGCCACAATCACAGAAGTAG-3′. This produced 610-bp and 848-bp amplicons from the wild-type and mutated alleles, respectively. For a representative panel of 12 differentially affected dogs (bold numbers, Table S1), the mutated amplicon encompassing the SINE was purified and sequenced (GATC, Germany). Passed sequences were aligned using the Multalin version 5.4.1 software (multalin.toulouse.inra.fr).

### SNPs Selection and Genotyping

These experimental steps were performed as previously described [42]. The list of the 15 selected SNPs with their positions is provided in Table S4. Primer sequences and optimal melting temperatures are detailed in Table S5.

### Haplotype Analysis and Clustering

Haplotype phases were inferred using the program fastPHASE version 1.4.0. Haplotype frequencies and associations were calculated using the statistical software package PLINK (http://pngu.mgh.harvard.edu/purcell/plink) (v1.07). Clustering analyses were performed using PLINK to compute the IBS distance matrice, and hierarchical clustering using the R function Hclust (http://cran.r-project.org/). Both PLINK and HCLUST use distance matrix to perform clustering. For HCLUST, we used a method that merged clusters based on a point biserial correlation with the Hubert Gamma statistics, that measures the correlation between groupings and distances.

## Supporting Information

Figure S1 **Size conservation of the SINE insertion in 25 unrelated affected Labradors.** Dogs were from the US (US; n = 20), the UK (UK; n = 3), Denmark (n = 1) and Canada (n = 1). In every tested dog, a unique band of the expected size is observed after the specific amplification of the SINE flanked by priming regions from exon 2.
(PDF)

Figure S2 **Identity of SINE sequences amplified from 12 affected Labradors.** Dogs (bolded in Table S1) were from the US (n = 7), Germany (n = 2), UK (n = 1), Denmark (n = 1) and France (n = 1; FR-2 is a founder dog of our experimental pedigree). The SINE sequence is shown in red, inserted within exon 2 of the *PTPLA* gene, which partial sequence is shown in green. The two 13-bp repeat sequences flanking the SINE are included in light-grey boxes.
(PDF)

Figure S3 **Haplotypes of 71 Labradors in a 9-Mb region around the** ***PTPLA*** **locus.** SNP positions (in kbp) from the centromeric to the telomeric end of the chromosome are listed on the left of charts. Dogs, identified by the two-letter code of their country followed by a unique incremental number for each country, are listed on the top of charts. For each dog, the two haplotypes are represented using a color code. The *PTPLA^cnm^* allele is represented by a black dot (•) and the wild-type *PTPLA^+^* allele by a “+”.
(PDF)

Table S1 **Summarized clinical signs, histopathological features and assigned genotypes of Labradors from the international confirmation panel.** Dogs were sorted by their country of origin and individually identified by the two-letter code of their country followed by a unique incremental number for each country. The 12 dogs for which SINE sequences have been assessed are bolded and shaded in grey. When available, the age at which biopsies were obtained is indicated. The initial diagnosis made by co-authors, who are qualified veterinarians (NO, JP, SB) or pathologists (GDS, TB), is reminded. When available, informative data excerpted from their pedigree are provided. In the last column, the assigned genotype at the *PTPLA* locus is given. Abbreviations: AF, Atrophic fibers; ARF, Anguloid-Round fibers; FatI, Fatty infiltration; FSV, Fiber size variation; GA, Gait abnormalities; InternN, Internalized nuclei; HypoT, hypotrophy; NF, Necrotic myofibers; NonInf, Non inflammatory; NR, Nemaline rods; no PR, no patellar reflex; type 1P, type 1 fiber predominance; WK, Weakness.
(PDF)

Table S2 **Numbers by genotype and sex of Labradors tested for medical or breeding purposes.** The period of testing was 2005–2012.
(PDF)

Table S3 **Numbers by genotype and coat colors of Labradors tested for medical or breeding purposes.** The period of testing was 2005–2012.
(PDF)

Table S4 **Positional information for the 15 polymorphic SNPs from CFA2 used in the haplotype analysis.** Their names and position, from the centromere of CFA2, are indicated in the two first columns. The first group from BICF2P407690 to BICF2P583542 encompasses the ∼4.2 Mb centromeric region of *PTPLA*. The second group from BICF2P642478 to BICF2S23249211 encompasses the ∼4.8 Mb telomeric region of *PTPLA*.
(PDF)

Table S5 **Experimental conditions to amplify the 15 polymorphic SNPs from CFA2 used in the haplotype analysis.** Primers listed were used to amplify the sequence (Forward and Reverse primers) and to identify the SNP (Sequencing primer).
(PDF)
