# Supplementary figures and images for: Centronuclear Myopathy in Labrador Retrievers: A Recent Founder Mutation in the PTPLA Gene Has Rapidly Disseminated Worldwide
Source: PLoS One. 2012 Oct 5;7(10):e46408. doi: 10.1371/journal.pone.0046408 (PMC3465307; doi:10.1371/journal.pone.0046408)

Figure S1

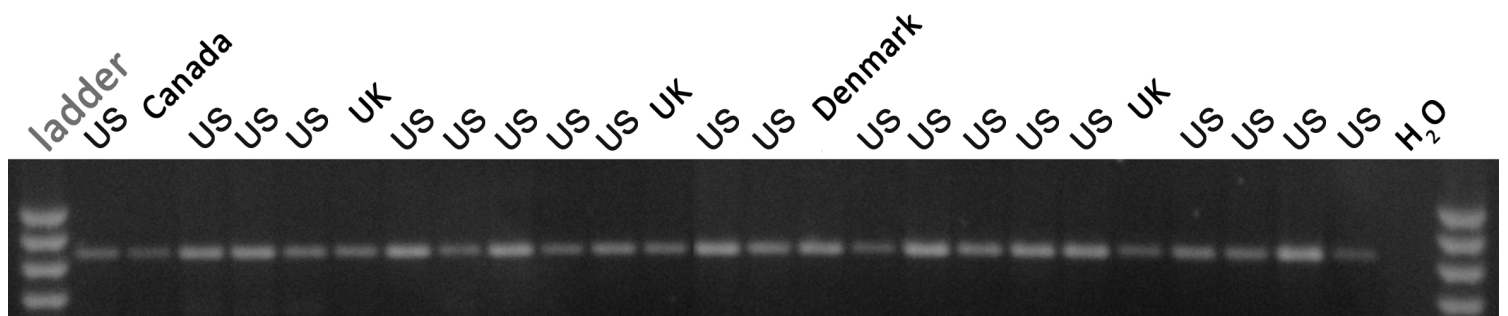

Supplement: Figure S1 — Size conservation of the SINE insertion in 25 unrelated affected Labradors. Dogs were from the US (US; n = 20), the UK (UK; n = 3), Denmark (n = 1) and Canada (n = 1). In every tested dog, a unique band of the expected size is observed after the specific amplification of the SINE flanked by priming regions from exon 2. (PDF) [file pone.0046408.s001.pdf]

Figure S3

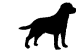

Healthy dogs

G A T C

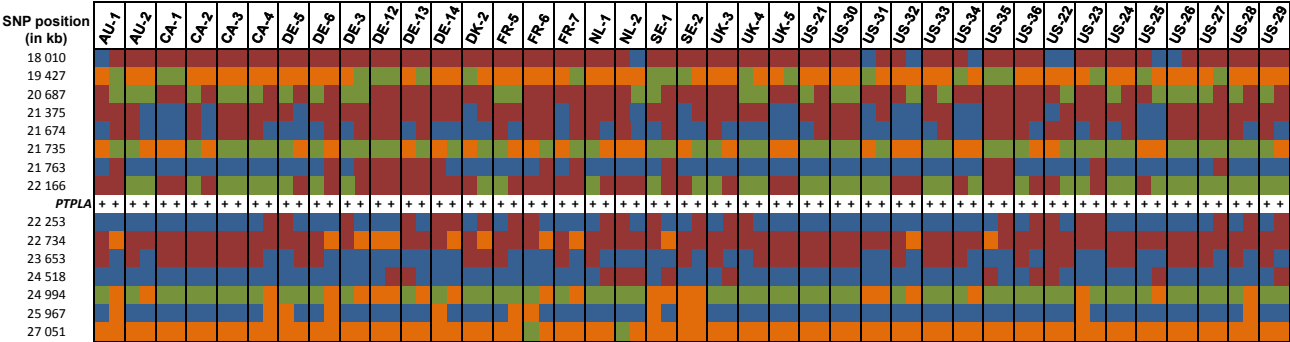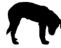

CNM dogs

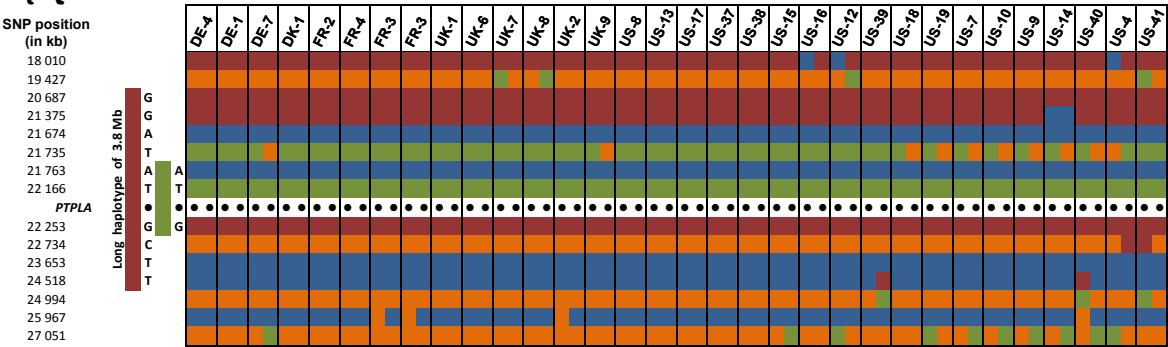

Supplement: Figure S3 — Haplotypes of 71 Labradors in a 9-Mb region around the PTPLA locus. SNP positions (in kbp) from the centromeric to the telomeric end of the chromosome are listed on the left of charts. Dogs, identified by the two-letter code of their country followed by a unique incremental number for each country, are listed on the top of charts. For each dog, the two haplotypes are represented using a color code. The PTPLAcnm allele is represented by a black dot (•) and the wild-type PTPLA+ allele by a “+”. (PDF) [file pone.0046408.s003.pdf]
