# Supplementary material for: Centronuclear Myopathy in Labrador Retrievers: A Recent Founder Mutation in the PTPLA Gene Has Rapidly Disseminated Worldwide
Source: PLoS One. 2012 Oct 5;7(10):e46408. doi: 10.1371/journal.pone.0046408 (PMC3465307; doi:10.1371/journal.pone.0046408)
Supplement: Figure S2 — Identity of SINE sequences amplified from 12 affected Labradors. Dogs (bolded in Table S1) were from the US (n = 7), Germany (n = 2), UK (n = 1), Denmark (n = 1) and France (n = 1; FR-2 is a founder dog of our experimental pedigree). The SINE sequence is shown in red, inserted within exon 2 of the PTPLA gene, which partial sequence is shown in green. The two 13-bp repeat sequences flanking the SINE are included in light-grey boxes. (PDF) [file pone.0046408.s002.pdf]

Figure S2

	1						70
US-8	CACACAAAGG	TTTTTTTTTT	TTTTTTAAAT	TTTTTTTTTT	AAATTTTTTT	TTTTTAATTT	TTTTTTATTT
US-9	CACACAAAGG	TTTTTTTTTT	TTTTTTAAAT	TTTTTTTTTT	AAATTTTTTT	TTTTTAATTT	TTTTTTATTT
US-10	CACACAAAGG	TTTTTTTTTT	TTTTTTAAAT	TTTTTTTTTT	AAATTTTTTT	TTTTTAATTT	TTTTTTATTT
US-13	CACACAAAGG	TTTTTTTTTT	TTTTTTAAAT	TTTTTTTTTT	AAATTTTTTT	TTTTTAATTT	TTTTTTATTT
US-14	CACACAAAGG	TTTTTTTTTT	TTTTTTAAAT	TTTTTTTTTT	AAATTTTTTT	TTTTTAATTT	TTTTTTATTT
US-15	CACACAAAGG	TTTTTTTTTT	TTTTTTAAAT	TTTTTTTTTT	AAATTTTTTT	TTTTTAATTT	TTTTTTATTT
US-16	CACACAAAGG	TTTTTTTTTT	TTTTTTAAAT	TTTTTTTTTT	AAATTTTTTT	TTTTTAATTT	TTTTTTATTT
DE-4	CACACAAAGG	TTTTTTTTTT	TTTTTTAAAT	TTTTTTTTTT	AAATTTTTTT	TTTTTAATTT	TTTTTTATTT
DE-7	CACACAAAGG	TTTTTTTTTT	TTTTTTAAAT	TTTTTTTTTT	AAATTTTTTT	TTTTTAATTT	TTTTTTATTT
UK-1	CACACAAAGG	TTTTTTTTTT	TTTTTTAAAT	TTTTTTTTTT	AAATTTTTTT	TTTTTAATTT	TTTTTTATTT
DK-1	CACACAAAGG	TTTTTTTTTT	TTTTTTAAAT	TTTTTTTTTT	AAATTTTTTT	TTTTTAATTT	TTTTTTATTT
FR-2	CACACAAAGG	TTTTTTTTTT	TTTTTTAAAT	TTTTTTTTTT	AAATTTTTTT	TTTTTAATTT	TTTTTTATTT

	71						140
US-8	ATTTATGATA	GTCACACACA	GATAGAGAGA	GAGGCAGAGA	CACAGGCAGA	GGGAGAAGCA	GGCTCCATGC
US-9	ATTTATGATA	GTCACACACA	GATAGAGAGA	GAGGCAGAGA	CACAGGCAGA	GGGAGAAGCA	GGCTCCATGC
US-10	ATTTATGATA	GTCACACACA	GATAGAGAGA	GAGGCAGAGA	CACAGGCAGA	GGGAGAAGCA	GGCTCCATGC
US-13	ATTTATGATA	GTCACACACA	GATAGAGAGA	GAGGCAGAGA	CACAGGCAGA	GGGAGAAGCA	GGCTCCATGC
US-14	ATTTATGATA	GTCACACACA	GATAGAGAGA	GAGGCAGAGA	CACAGGCAGA	GGGAGAAGCA	GGCTCCATGC
US-15	ATTTATGATA	GTCACACACA	GATAGAGAGA	GAGGCAGAGA	CACAGGCAGA	GGGAGAAGCA	GGCTCCATGC
US-16	ATTTATGATA	GTCACACACA	GATAGAGAGA	GAGGCAGAGA	CACAGGCAGA	GGGAGAAGCA	GGCTCCATGC
DE-4	ATTTATGATA	GTCACACACA	GATAGAGAGA	GAGGCAGAGA	CACAGGCAGA	GGGAGAAGCA	GGCTCCATGC
DE-7	ATTTATGATA	GTCACACACA	GATAGAGAGA	GAGGCAGAGA	CACAGGCAGA	GGGAGAAGCA	GGCTCCATGC
UK-1	ATTTATGATA	GTCACACACA	GATAGAGAGA	GAGGCAGAGA	CACAGGCAGA	GGGAGAAGCA	GGCTCCATGC
DK-1	ATTTATGATA	GTCACACACA	GATAGAGAGA	GAGGCAGAGA	CACAGGCAGA	GGGAGAAGCA	GGCTCCATGC
FR-2	ATTTATGATA	GTCACACACA	GATAGAGAGA	GAGGCAGAGA	CACAGGCAGA	GGGAGAAGCA	GGCTCCATGC

	141						210
US-8	ACCGGGAGCC	CGACGTGGGA	CTCGATCCCG	GGTCTCCAGG	ATCGCGCCCT	GGGCCAAAGG	CAGGCGCCAA
US-9	ACCGGGAGCC	CGACGTGGGA	CTCGATCCCG	GGTCTCCAGG	ATCGCGCCCT	GGGCCAAAGG	CAGGCGCCAA
US-10	ACCGGGAGCC	CGACGTGGGA	CTCGATCCCG	GGTCTCCAGG	ATCGCGCCCT	GGGCCAAAGG	CAGGCGCCAA
US-13	ACCGGGAGCC	CGACGTGGGA	CTCGATCCCG	GGTCTCCAGG	ATCGCGCCCT	GGGCCAAAGG	CAGGCGCCAA
US-14	ACCGGGAGCC	CGACGTGGGA	CTCGATCCCG	GGTCTCCAGG	ATCGCGCCCT	GGGCCAAAGG	CAGGCGCCAA
US-15	ACCGGGAGCC	CGACGTGGGA	CTCGATCCCG	GGTCTCCAGG	ATCGCGCCCT	GGGCCAAAGG	CAGGCGCCAA
US-16	ACCGGGAGCC	CGACGTGGGA	CTCGATCCCG	GGTCTCCAGG	ATCGCGCCCT	GGGCCAAAGG	CAGGCGCCAA
DE-4	ACCGGGAGCC	CGACGTGGGA	CTCGATCCCG	GGTCTCCAGG	ATCGCGCCCT	GGGCCAAAGG	CAGGCGCCAA
DE-7	ACCGGGAGCC	CGACGTGGGA	CTCGATCCCG	GGTCTCCAGG	ATCGCGCCCT	GGGCCAAAGG	CAGGCGCCAA
UK-1	ACCGGGAGCC	CGACGTGGGA	CTCGATCCCG	GGTCTCCAGG	ATCGCGCCCT	GGGCCAAAGG	CAGGCGCCAA
DK-1	ACCGGGAGCC	CGACGTGGGA	CTCGATCCCG	GGTCTCCAGG	ATCGCGCCCT	GGGCCAAAGG	CAGGCGCCAA
FR-2	ACCGGGAGCC	CGACGTGGGA	CTCGATCCCG	GGTCTCCAGG	ATCGCGCCCT	GGGCCAAAGG	CAGGCGCCAA

	211				254
US-8	ACCGCTGCGC	CACCCAGGGA	TCCCCCACAC	AAAGGTTTAT	ATAA
US-9	ACCGCTGCGC	CACCCAGGGA	TCCCCCACAC	AAAGGTTTAT	ATAA
US-10	ACCGCTGCGC	CACCCAGGGA	TCCCCCACAC	AAAGGTTTAT	ATAA
US-13	ACCGCTGCGC	CACCCAGGGA	TCCCCCACAC	AAAGGTTTAT	ATAA
US-14	ACCGCTGCGC	CACCCAGGGA	TCCCCCACAC	AAAGGTTTAT	ATAA
US-15	ACCGCTGCGC	CACCCAGGGA	TCCCCCACAC	AAAGGTTTAT	ATAA
US-16	ACCGCTGCGC	CACCCAGGGA	TCCCCCACAC	AAAGGTTTAT	ATAA
DE-4	ACCGCTGCGC	CACCCAGGGA	TCCCCCACAC	AAAGGTTTAT	ATAA
DE-7	ACCGCTGCGC	CACCCAGGGA	TCCCCCACAC	AAAGGTTTAT	ATAA
UK-1	ACCGCTGCGC	CACCCAGGGA	TCCCCCACAC	AAAGGTTTAT	ATAA
DK-1	ACCGCTGCGC	CACCCAGGGA	TCCCCCACAC	AAAGGTTTAT	ATAA
FR-2	ACCGCTGCGC	CACCCAGGGA	TCCCCCACAC	AAAGGTTTAT	ATAA
