# Supplementary material for: Centronuclear Myopathy in Labrador Retrievers: A Recent Founder Mutation in the PTPLA Gene Has Rapidly Disseminated Worldwide
Source: PLoS One. 2012 Oct 5;7(10):e46408. doi: 10.1371/journal.pone.0046408 (PMC3465307; doi:10.1371/journal.pone.0046408)
Supplement: Table S1 — Summarized clinical signs, histopathological features and assigned genotypes of Labradors from the international confirmation panel. Dogs were sorted by their country of origin and individually identified by the two-letter code of their country followed by a unique incremental number for each country. The 12 dogs for which SINE sequences have been assessed are bolded and shaded in grey. When available, the age at which biopsies were obtained is indicated. The initial diagnosis made by co-authors, who are qualified veterinarians (NO, JP, SB) or pathologists (GDS, TB), is reminded. When available, informative data excerpted from their pedigree are provided. In the last column, the assigned genotype at the PTPLA locus is given. Abbreviations: AF, Atrophic fibers; ARF, Anguloid-Round fibers; FatI, Fatty infiltration; FSV, Fiber size variation; GA, Gait abnormalities; InternN, Internalized nuclei; HypoT, hypotrophy; NF, Necrotic myofibers; NonInf, Non inflammatory; NR, Nemaline rods; no PR, no patellar reflex; type 1P, type 1 fiber predominance; WK, Weakness. (PDF) [file pone.0046408.s004.pdf]

Table S1

# Dog	Country	Unique Identifier	Clinical report	Age of biopsy (months)	Histopathological features	Diagnosis	Pedigre information	Genotype (*PTPLA* locus)
1	USA	US-3	WK, HypoT		-	HMLR	Compatible with AR transmission (see Fig. 2B)	**cnm/cnm**
2	USA	US-7	-	5	AF, ARF, rare NF	Neuromyopathy group of HMLR	-	**cnm/cnm**
3	USA	**US-8**	GA, Cervical WK, HypoT, no PR	2	Neurogenic atrophy	HMLR	-	**cnm/cnm**
4	USA	**US-9**	-	3	AF, fibrosis, FatI, "Wallerian-like" degeneration, NR	Chronic nonInf myopathy, early neuropathy	-	**cnm/cnm**
5	USA	**US-10**	-	-	AF, ARF, FSV, type 1P, rare NF	NonInf myopathy consistent with HMLR	-	**cnm/cnm**
6	USA	US-11	-	-	type 1P, no other abnormality	HMLR	-	**+/cnm**
7	USA	US-12	-	7	AF, ARF, FSV	NonInf myopathy consistent with HMLR	-	**cnm/cnm**
8	USA	**US-13**	-	6	AF, ARF, FSV, type 1P, several NF	NonInf myopathy consistent with HMLR	-	**cnm/cnm**
9	USA	**US-14**	GA, WK, HypoT, no PR, abnormal EMG	12	FSV, rare NF	NonInf myopathy consistent with HMLR	-	**cnm/cnm**
10	USA	**US-15**	-	4	AF, FSV	NonInf myopathy consistent with HMLR	-	**cnm/cnm**
11	USA	**US-16**	-	7	AF, ARF, FSV, FatI	NonInf myopathy consistent with HMLR	-	**cnm/cnm**
12	USA	US-17	-	6	ARF, FSV, type 1P	NonInf myopathy consistent with HMLR	-	**cnm/cnm**
13	USA	US-18	-	4	AF, ARF, FSV, type 1P	NonInf myopathy consistent with HMLR	-	**cnm/cnm**
14	USA	US-19	-	7	AF, ARF, FSV	Neuromyopathy group of HMLR	-	**cnm/cnm**
15	USA	US-20	GA, WK, no PR	3	AF, FSV	HMLR	-	**cnm/cnm**
16	Germany	DE-1	Severely affected, fibrillation potentials, post-stimulations waves	-	FSV, NF, no atrophy, type 1P	Labrador myopathy	Swiss pedigree	**cnm/cnm**
17	Germany	DE-2		5	ARF, FSV, NF, focal atrophy, type 1P	HMLR		**cnm/cnm**
18	Germany	DE-3	Traumatic lesion of the plexus brachialis	-		Neuropathy	-	**+/+**
19	Germany	**DE-4**	Apathy, low appetite, HypoT	5	ARF, FSV, NF, focal atrophy in type I and II fibers	Chronic necrotic myopathy consistent with HMLR	Grandfather: UK champion Great-grandparents: Four out of 8 were UK champions	**cnm/cnm**
20	Germany	DE-5	WK, symptoms of myasthenia gravis	16	Few ARF, light FSV, NF no InternN	Chronic neuromyopathy	Grandparents: Belgian and Swedish champions Great-grandparents: Belgian and Norvegian champions Great-great-grandparents: US, UK, Swedish and Norvegian champions	**+/+**
21	Germany	DE-6	**-**	-	Focal neurogenic lesions	Neuromyopathy	-	**+/+**
22	Germany	**DE-7**	Affected	-		Muscular dystrophy	-	**cnm/cnm**
23	Germany	DE-8	Normal	-	-	Labrador myopathy	Swiss pedigree	**+/+**
24	Germany	DE-9	Normal	-	-	Labrador myopathy	Swiss pedigree	**+/+**
25	Germany	DE-10	Affected	-		Labrador myopathy	-	**+/+**
26	Germany	DE-11	Lameness	-		Labrador myopathy	-	**+/+**
27	UK	**UK-1**	-			HMLR	-	**cnm/cnm**
28	UK	UK-2	-			HMLR	-	**cnm/cnm**
29	UK	UK-3	-			HMLR	-	**+/+**
30	Denmark	**DK-1**	Low weight, no PR	4	-	HMLR	-	**cnm/cnm**
31	France	**FR-2**	GA, Cervical WK, fatigability, HypoT, hypotonicity, no PR	5	AF, ARF, FSV, NF, type 1P, few InternN, FatI	CNM	Father: UK champion Paternal grandparents: UK Great-grandparents: All (8/8) from UK	**cnm/cnm**
32	France	FR-3	Fatigability	5	AF, FSV, type 1P, few InternN	CNM	-	**cnm/cnm**
