# Supplementary material for: Centronuclear Myopathy in Labrador Retrievers: A Recent Founder Mutation in the PTPLA Gene Has Rapidly Disseminated Worldwide
Source: PLoS One. 2012 Oct 5;7(10):e46408. doi: 10.1371/journal.pone.0046408 (PMC3465307; doi:10.1371/journal.pone.0046408)
Supplement: Table S2 — Numbers by genotype and sex of Labradors tested for medical or breeding purposes. The period of testing was 2005–2012. (PDF) [file pone.0046408.s005.pdf]

Table S2

	+/+	+/cnm	cnm/cnm	Total
**Male**	**2 516**<br>*82.1%*	**513**<br>*16.7%*	**35**<br>*1.1%*	3 064
**Female**	**3 657**<br>*83.8%*	**660**<br>*15.1%*	**45**<br>*1.0%*	4 362
**Total**	6 173	1 173	80	**7 426**
