# Supplementary material for: Centronuclear Myopathy in Labrador Retrievers: A Recent Founder Mutation in the PTPLA Gene Has Rapidly Disseminated Worldwide
Source: PLoS One. 2012 Oct 5;7(10):e46408. doi: 10.1371/journal.pone.0046408 (PMC3465307; doi:10.1371/journal.pone.0046408)
Supplement: Table S3 — Numbers by genotype and coat colors of Labradors tested for medical or breeding purposes. The period of testing was 2005–2012. (PDF) [file pone.0046408.s006.pdf]

Table S3

	+/+	+/cnm	cnm/cnm	Total
**Black**	**3 604**	**771**	**39**	4 414
	81.6%	*17.4%*	*0.9%*	
**Yellow**	**1 682**	**219**	**21**	1 922
	87.6%	*11.4%*	*1.1%*	
**Chocolate**	**848**	**171**	**17**	1 036
	81.9%	*16.6%*	*1.6%*	
**Unknown**	**39**	**12**	**3**	54
**Total**	6 173	1 173	80	**7 426**
