# Supplementary material for: Centronuclear Myopathy in Labrador Retrievers: A Recent Founder Mutation in the PTPLA Gene Has Rapidly Disseminated Worldwide
Source: PLoS One. 2012 Oct 5;7(10):e46408. doi: 10.1371/journal.pone.0046408 (PMC3465307; doi:10.1371/journal.pone.0046408)
Supplement: Table S4 — Positional information for the 15 polymorphic SNPs from CFA2 used in the haplotype analysis. Their names and position, from the centromere of CFA2, are indicated in the two first columns. The first group from BICF2P407690 to BICF2P583542 encompasses the ∼4.2 Mb centromeric region of PTPLA. The second group from BICF2P642478 to BICF2S23249211 encompasses the ∼4.8 Mb telomeric region of PTPLA. (PDF) [file pone.0046408.s007.pdf]

Table S4

CFA2: 18.0-22.16 Mb

SNP_Id	Position bp	Name on Figures	Distance between SNPs	Cumulated distance from PTPLA to centromere	SNP variation
BICF2P407690	18 009 723	18010	1 417 040	4 185 659	[A/G]
BICF2S23117062	19 426 763	19427	1 259 933	2 768 619	[A/G]
BICF2P768278	20 686 696	20687	688 617	1 508 686	[C/A]
BICF2S23334088	21 375 313	21375	298 510	820 069	[C/T]
BICF2S23256430	21 673 823	21674	60 774	521 559	[C/T]
BICF2S23717225	21 734 597	21735	28 755	460 785	[T/C]
BICF2P375846	21 763 352	21763	402 360	432 030	[T/C]
BICF2P583542	22 165 712	22166	0	29 670	[G/T]

PTPLA: 22.19-22.22 Mb

CFA2: 22.25-27.05 Mb

SNP_Id	Position bp	Name on Figures	Distance between SNPs	Cumulated distance from PTPLA to telomere	SNP variation
BICF2P642478	22 252 747	22253	0	36 911	[T/C]
BICF2S23136859	22 733 549	22734	480 802	517 713	[C/G]
BICF2S23329178	23 652 879	23653	919 330	1 437 043	[A/G]
BICF2S23351046	24 518 409	24518	865 530	2 302 573	[T/C]
BICF2S23335689	24 994 400	24994	475 991	2 778 564	[C/T]
BICF2S2361673	25 966 952	25967	972 552	3 751 116	[C/A]
BICF2S23249211	27 051 266	27051	1 084 314	4 835 430	[A/G]
