# Supplementary material for: Centronuclear Myopathy in Labrador Retrievers: A Recent Founder Mutation in the PTPLA Gene Has Rapidly Disseminated Worldwide
Source: PLoS One. 2012 Oct 5;7(10):e46408. doi: 10.1371/journal.pone.0046408 (PMC3465307; doi:10.1371/journal.pone.0046408)
Supplement: Table S5 — Experimental conditions to amplify the 15 polymorphic SNPs from CFA2 used in the haplotype analysis. Primers listed were used to amplify the sequence (Forward and Reverse primers) and to identify the SNP (Sequencing primer). (PDF) [file pone.0046408.s008.pdf]

Table S5

CFA2: 18.0-22.16 Mb

SNP_Id	Names on Figures	Forward primer 5'-3'	Reverse primer 5'-3'	Tm	Sequencing primer 5'-3'
BICF2P407690	18010	GAGCCTTTTCTCTTCTGTCAGAGT	CCAGCTCCCAGATTCCTTGTT	60°C	TTACATTCAGAAGCTAGGAT
BICF2S23117062	19427	CCTAAGGGAGATTACTGACTGAGC	TTTACAAAGCCACAGAGGAGACAT	60°C	CACAGAGGAGACATCAAGT
BICF2P768278	20687	CCGTTTTCTTCCCCATTCA	AGAGTTCTCACTGTGCCATCAC	63,5°C	CCTTCACCATTTCCCT
BICF2S23334088	21375	TGTAGCTTTTTGTAAGCCATCATC	TCCATTTAGCCATGGTCTTCATT	55°C	CCTCCCATGAGCACT
BICF2S23256430	21674	ATCAGAACAGGCACCTGGTATTT	AAATGGCCTTTCCCACTAACA	63,5°C	CCCACTAACACACAGATTG
BICF2S23717225	21735	TCCCCAAAGGCGGTTTAAAT	GGGGTGTGGATTGCAAAAG	55°C	CAAAGGCGGTTTAAAT
BICF2P375846	21763	GGTACATGATTTTGTCACCCTAGA	GGGTTCAATTTGTTCTTGAGAAAG	63,5°C	TTGATTATCAATAATAACAG
BICF2P583542	22166	GGCTTATTACCGTAGCAAAGTATG	GTTTACCTTGTTCACCTGTTCAGT	63,5°C	TGATCTCACAGTTCTTGAGT

CFA2: 22.25-27.05 Mb

SNP_Id	Names on Figures	Forward primer 5'-3'	Reverse primer 5'-3'	Tm	Sequencing primer 5'-3'
BICF2P642478	22253	GGGGCAGGACAGGTAAGTTTTC	TCCACCACTAGATCCCAGATATTC	61°C	CCTATCCAAGCAGAGTAAT
BICF2S23136859	22734	GCTATAGTCAGCTTGGGGCTTATT	AACATCCCCCCACAAACC	55°C	GGTTTTGTGGAACATGG
BICF2S23329178	23653	TGAGAGGACAAAAGGGTGGATAG	AAGCCTGTCTTTTGGCACTTTA	55°C	GGTGGATAGTCCAGACTACA
BICF2S23351046	24518	GGCAGCGAGAGGCCAACT	GTCACGCGTTGCTAGGAGAC	63,5°C	TGCTAGGAGACGCGA
BICF2S23335689	24994	GTGCTTCATTTATTTGTGGACTTT	TTTATGATTAGACCACCCTTGACA	55°C	GGTTTTCACCTAGCCTG
BICF2S2361673	25967	TTTCTGCGGGACACAGCT	CTGCTACGGTCTCTTACAGATTCG	55°C	TGAGCTATGAGCACACTG
BICF2S23249211	27051	TCCATCAGGCATAGCTACGTT	TGACCAAAGCTCAGGAGATTTC	55°C	AAGCTCAGGAGATTTCA
